# Translocation Breakpoints Preferentially Occur in Euchromatin and Acrocentric Chromosomes

**DOI:** 10.3390/cancers10010013

**Published:** 2018-01-08

**Authors:** Cheng-Yu Lin, Ankit Shukla, John P. Grady, J. Lynn Fink, Eloise Dray, Pascal H.G. Duijf

**Affiliations:** 1 University of Queensland Diamantina Institute, The University of Queensland, Translational Research Institute, 37 Kent Street, Brisbane, QLD 4102, Australia; c.lin2@uq.edu.au (C.-Y.L.); ankit.shukla@uq.edu.au (A.S.); j.grady@garvan.org.au (J.P.G.); l.fink@uq.edu.au (J.L.F.); 2 Institute of Health and Biomedical Innovation, Queensland University of Technology, Translational Research Institute, 37 Kent Street, Brisbane, QLD 4102, Australia; eloise.dray@qut.edu.au; 3 Mater Research Institute-The University of Queensland, Translational Research Institute, 37 Kent Street, Brisbane, QLD 4102, Australia

**Keywords:** translocations, DNA double strand breaks, DNA repair, leukemia, lymphoma, V(D)J recombination, CTCF, cohesin, nucleolus, acrocentric chromosomes

## Abstract

Chromosomal translocations drive the development of many hematological and some solid cancers. Several factors have been identified to explain the non-random occurrence of translocation breakpoints in the genome. These include chromatin density, gene density and CCCTC-binding factor (CTCF)/cohesin binding site density. However, such factors are at least partially interdependent. Using 13,844 and 1563 karyotypes from human blood and solid cancers, respectively, our multiple regression analysis only identified chromatin density as the primary statistically significant predictor. Specifically, translocation breakpoints preferentially occur in open chromatin. Also, blood and solid tumors show markedly distinct translocation signatures. Strikingly, translocation breakpoints occur significantly more frequently in acrocentric chromosomes than in non-acrocentric chromosomes. Thus, translocations are probably often generated around nucleoli in the inner nucleoplasm, away from the nuclear envelope. Importantly, our findings remain true both in multivariate analyses and after removal of highly recurrent translocations. Finally, we applied pairwise probabilistic co-occurrence modeling. In addition to well-known highly prevalent translocations, such as those resulting in *BCR*-*ABL1* (*BCR*-*ABL*) and *RUNX1*-*RUNX1T1* (*AML1*-*ETO*) fusion genes, we identified significantly underrepresented translocations with putative fusion genes, which are probably subject to strong negative selection during tumor evolution. Taken together, our findings provide novel insights into the generation and selection of translocations during cancer development.

## 1. Introduction

Chromosome instability (CIN) is a hallmark of cancer [1,2]. It refers to an increased gain of chromosomal abnormalities. CIN typically predicts poor cancer patient survival and increased drug-resistance [2,3,4]. Numerical CIN (n-CIN) comprises the gain or loss of whole chromosomes and leads to aneuploidy. N-CIN is common in solid tumors [5] and is often caused by aberrant expression of cell cycle, centrosome or centromere proteins, which in turn leads to centrosome amplification or mitotic aberrations followed by chromosome missegregation [6,7,8,9,10]. Structural CIN (s-CIN) refers to the gain, loss or rearrangement of fractions of chromosomes. Copy number changes can be focal or involve large chromosomal segments, such as entire chromosome arms. Translocations are a form of chromosomal rearrangements, which are particularly common in leukemias, lymphomas and some sarcomas [11,12,13,14,15]. Translocations involve the rearrangement between heterologous chromosomes. This can promote tumorigenesis by altering gene expression, either via the generation of oncogenic fusion genes or by changing the location of regulatory elements and affecting the expression of oncogenes or tumor suppressor genes [11,15,16,17]. Examples include *RUNX1*-*RUNX1T1* (formerly *AML1*-*ETO*) fusion genes, which are the result of common t(8;21)(q22;q22) translocations in acute myeloid leukemia (AML) patients [16] and *BCR*-*ABL1* fusion genes caused by t(9;22)(q34;q11) translocations in chronic myeloid leukemia (CML) patients [17].

Translocations require the formation and resolution of DNA double strand breaks (DSBs). There are a number of mechanisms of DSB repair [18]. A study showed that abnormal kinetochore-microtubule attachments induce the formation of anaphase bridges during mitotic chromosome segregation, thereby inducing DSBs on chromosome arms and promoting translocations [19].

Interestingly, translocations do not occur randomly in the genome; they seem to preferentially occur at specific chromosomal locations. Gene density or chromatin density may predict the location of translocation breakpoints. Euchromatin is a loosely packed, gene-rich form of chromatin. Euchromatin facilitates DNA accessibility for gene regulatory proteins and RNA polymerase, and hence enables transcription. In contrast, heterochromatin is a tightly packed form of chromatin with repetitive sequences and is typically poor in genes [20]. It is mainly located near centromeres and telomeres. Euchromatin may be more susceptible to DSBs and hence translocation breakpoints.

V(D)J recombination—between variable (V), diversity (D) and joining (J) genes—maintains diversity of antibodies and T-cell receptors in B- and T-lymphocyte development and has been shown to promote some translocations [21]. The recombination activating gene (RAG) complex recognizes specific DNA sequences, named recombination signal sequences (RSS), to induce nicks for V, D, J gene rearrangement [22]. The nicks could later convert to DSBs, which are repaired by homologous recombination or non-homologous end joining [23]. Nonetheless, if nicks occur on other cryptic RSS elements rather than in the V(D)J gene locus [24,25], or DNA repair is defective [26], DSBs may subsequently lead to translocations. Importantly, V(D)J recombination requires the formation of chromatin-loops, which are established by CCCTC-binding factor (CTCF) [27,28].

CTCF, which co-localizes and cooperates with cohesin proteins [29,30,31], is also an essential and versatile regulator of gene expression [32]. CTCF/cohesin complexes act as insulators, preventing inactive genes from erroneous activation by enhancers. They do so by forming three-dimensional loops, which block enhancer-promoter contacts. CTCF/cohesin also provides a barrier for abnormal repressive heterochromatin spreading into vicinity domains [32]. Loss of CTCF/cohesin function has been shown to influence normal development. For example, deletion of CTCF/cohesin-binding sites on the lgH recombination regulatory region affects B-cell development [27]. Using array painting, Howarth et al. discovered that chromosomal translocation breakpoints correlate with DNA breaks of CTCF/cohesin sites in breast cancer cell lines [33].

Here, we used 15,407 human cancer karyotypes to investigate associations between chromosomal translocations and various parameters that could affect breakpoint frequency. We find that translocations preferentially occur in longer cytogenetic chromosome bands (i.e., chromosome regions that are distinctly recognizable under the microscope using cytogenetic stains, such as Giemsa [34]), euchromatin and regions rich in CTCF/cohesin binding sites or genes. However, multiple regression analysis identifies chromatin density as the major predictor. In addition, human chromosomes can be categorized into three cytogenetically distinct types: acrocentric, metacentric and submetacentric, depending on the position of the centromere in the chromosome. Interestingly, translocation breakpoints preferentially occur in acrocentric chromosomes, suggesting that translocations are often generated around nucleoli. Finally, probabilistic co-occurrence modeling provides new insights relevant to the selection of translocations during cancer development.

## 2. Results

### 2.1. Translocation Breakpoints Preferentially Occur in Longer Cytogenetic Chromosome Bands

**Figure 1:**
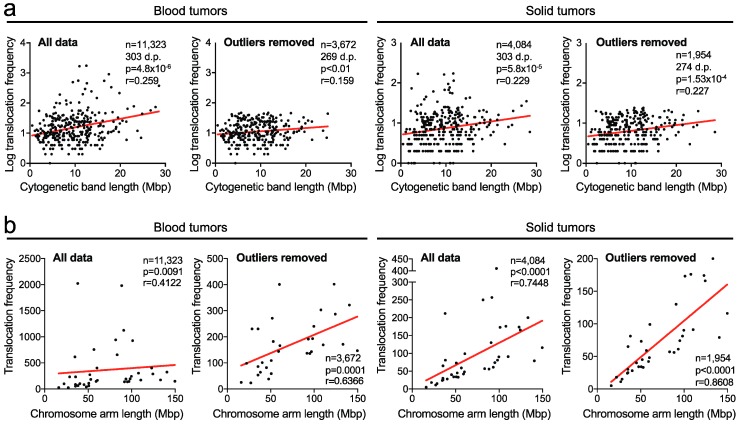
Translocation breakpoints preferentially occur in longer cytogenetic chromosome bands and arms. (**a**) Scatter plots of the cytogenetic chromosome band length in mega-base pairs (Mbp) and translocation frequencies within these bands in blood and solid tumors. Shown are analyses including all data, as well as analyses with data from which statistically identified outliers were removed (see main text). The latter was done to rule out the possibility that statistical significance was reached solely due to one or several highly frequent events which could skew the analyses. *p* and *r* values: Spearman correlations. D.p., data points. (**b**) Scatter plots as in (**a**) but at chromosome arm level.

Using quality-filtered karyotypes from 13,844 human blood cancers and 1563 human solid tumors [35], we considered all chromosomal breakpoints that resulted in a translocation and analyzed the rate at which translocation breakpoints occur in each cytogenetic chromosome band. We first tested whether there is a correlation between translocation frequency in a cytogenetic band and that band’s physical length. Not surprisingly, this revealed that breakpoints preferentially occur in longer cytogenetic bands in both blood and solid tumors (*r* = 0.259, *p* < 0.0001 and *r* = 0.229, *p* < 0.0001, respectively, Spearman’s correlation tests; Figure 1a).

As some specific translocations may occur at extremely high frequencies, they could introduce a bias in the analyses and heavily skew the outcome. To address this possibility, we used a method that combines robust regression and outlier removal, ROUT [36], to identify outliers. However, after removal of the identified outliers (at a false-discovery rate (FDR) of *q* = 0.01), the correlations remained highly statistically significant (*p* < 0.01 and *p* < 0.001, respectively; Figure 1a).

Similarly, we observed strong correlations between chromosome arm length and the translocation frequencies in both blood and solid cancers, irrespective of whether identified outliers were removed (all *p*-values < 0.01) (Figure 1b). Together, these data indicate that there is a strong positive association between the translocation breakpoint frequency and the length of the cytogenetic band or chromosome arm in which the breakpoint occurs.

### 2.2. Translocation Breakpoints Preferentially Occur in Open Chromatin

**Figure 2:**
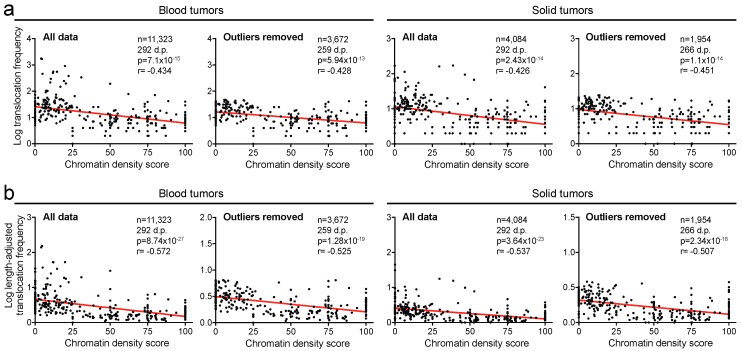
Translocation breakpoints preferentially occur in euchromatin. (**a**) Scatter plots of chromatin density score of cytogenetic chromosome bands correlated with translocation frequencies within these bands in blood and solid tumors. Analyses are also shown after outlier removal. (**b**) Scatter plots of chromatin density score of cytogenetic chromosome bands correlated with length-adjusted translocation frequencies of the bands in blood and solid tumors. D.p., data points. Statistics: see Figure 1.

Next, we assessed whether translocations are more likely to occur in open chromatin (euchromatin) or densely packed, “closed” chromatin (heterochromatin). To investigate that, we assigned an average chromatin density (ACD) score to each cytogenetic band. On a scale of 0 to 100, the ACD score indicates how loosely or tightly the chromatin is packed. Cytogenetic bands that were entirely open—euchromatic—received a score of 0, while bands whose chromatin was completely closed—heterochromatic—received a score of 100. For each cytogenetic band, the ACD score was calculated (see Methods). We independently plotted the translocation frequency of chromosome bands against their ACD scores. This showed that lower ACD scores are statistically significantly associated with higher frequencies of translocation breakpoints (*r* = −0.434, *p* < 0.0001 in blood tumors; *r* = −0.426, *p* < 0.0001 in solid tumors, Spearman’s correlation tests; Figure 2a). These correlations also remained statistically significant after removal of outliers identified by the ROUT method (at FDR *q* = 0.01; *r* = −0.428, *p* < 0.0001 in blood tumors; *r* = −0.451, *p* < 0.0001 in solid tumors; Figure 2a). Thus, these analyses indicate that translocation breakpoints are more likely to occur in loosely packed chromatin than in dense chromatin.

.

Importantly, our analyses above indicated that breakpoints more frequently occur in longer cytogenetic bands (Figure 1a). Therefore, to account for this, we normalized breakpoint frequencies to the length of the cytogenetic bands. To achieve that, we divided the breakpoint frequency of each band by its proportion of the whole genome length. We then plotted length-adjusted breakpoint frequencies of each band against their ACD scores for the blood or solid tumor cohorts. The relationships between length-adjusted breakpoint frequencies and ACD scores were stronger than for non-length-adjusted frequencies for both the blood and solid cancer cohorts (*r* = −0.572, *p* < 0.0001 and *r* = −0.537, *p* < 0.0001, respectively, Spearman’s correlation tests; Figure 2b). In addition, these remained highly significant after exclusion of outliers (*r* = −0.525, *p* < 0.0001 and *r* = −0.507, *p* < 0.0001, respectively; Figure 2b). Taken together, we conclude that translocation breakpoints preferentially occur in more open, euchromatic chromatin.

### 2.3. Translocation Breakpoints Preferentially Occur in Regions Rich in CTCF/Cohesin Binding Sites

**Figure 3:**
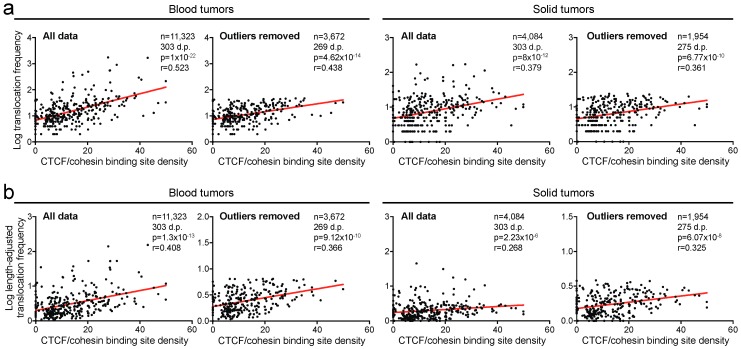
Translocation breakpoints preferentially occur in regions rich in CCCTC-binding factor (CTCF)/cohesin binding sites. (**a**) Scatter plots of CTCF/cohesin binding site densities in cytogenetic chromosome bands correlated with translocation frequencies within these bands in blood and solid tumors. Analyses after outlier removal are also shown. (**b**) Scatter plots of blood and solid tumors as in (a) but with translocation frequencies adjusted to the length of the bands in which they occur. D.p., data points. Statistics: see Figure 1.

We next asked whether translocation breakpoints might be more common in regions in which gene regulation occurs. As a surrogate for this, for each cytogenetic band, we calculated the density of the DNA binding sites for CTCF/cohesin, a DNA-binding protein complex that regulates transcription [28]. We found a significant correlation between breakpoint frequency and CTCF/cohesin binding site density for both blood and solid tumors (*r* = 0.523, *p* < 0.0001 and *r* = 0.379, *p* < 0.0001, respectively, Spearman’s correlation tests; Figure 3a). Removal of outliers only marginally affected the strength of the correlations between these two parameters (*r* = 0.438, *p* < 0.0001 and *r* = 0.361, *p* < 0.0001, respectively, Spearman’s correlation tests; Figure 3a).

Additionally, we independently plotted the length-adjusted breakpoint frequencies against the CTCF/cohesin binding site densities of the cytogenetic bands. These analyses indicate that translocation breakpoints are more likely to occur in regions rich in CTCF/cohesin binding sites. (*r* = 0.408, *p* < 0.0001 for blood tumors; *r* = 0.268, *p* < 0.0001 for solid tumors; Figure 3b). Similar to previous analyses, the strength of the correlations was only slightly affected after removal of outliers (*r* = 0.366, *p* < 0.0001 and *r* = 0.325, *p* < 0.0001, respectively, Spearman’s correlation tests, Figure 3b). Thus, translocation breakpoints preferentially occur in regions of gene regulation.

### 2.4. Translocation Breakpoints Preferentially Occur in Gene-Rich Regions

**Figure 4:**
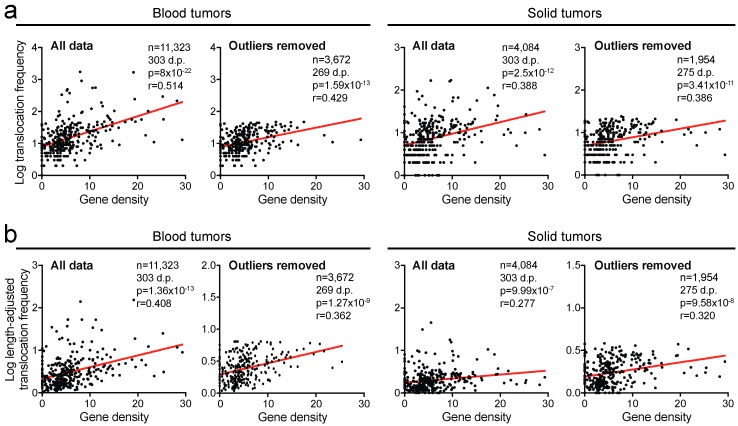
Translocation breakpoints preferentially occur in gene-rich regions. (**a**) Scatter plots of gene densities in cytogenetic chromosome bands correlated with translocation frequencies within these bands in blood and solid tumors. (**b**) Scatter plots of gene densities in cytogenetic chromosome bands correlated with length-adjusted translocation frequencies in blood and solid tumors. D.p., data points. Statistics: see Figure 1.

We next asked whether there is a relationship between the translocation rate and gene density. Direct comparison of these parameters showed that they strongly correlate positively in both blood tumors and solid tumors (*r* = 0.514, *p* < 0.0001 and *r* = 0.388, *p* < 0.0001, respectively, Spearman’s correlation tests; Figure 4a) and removal of outliers only slightly affected the strength of these correlations (*r* = 0.429, *p* < 0.0001 and *r* = 0.386, *p* < 0.0001, Spearman’s correlation tests; Figure 4a). Similarly, a significant association was observed after length-adjustment (*r* = 0.408, *p* < 0.0001 in blood tumors; *r* = 0.277, *p* < 0.0001 in solid tumors, Spearman’s correlation tests; Figure 4b) and this remained significant after outliers were removed (*r* = 0.362, *p* < 0.0001 and *r* = 0.320, *p* < 0.0001, respectively, Spearman’s correlation tests; Figure 4b). This indicates that translocation breakpoints preferentially occur in gene-rich regions.

### 2.5. Chromatin Density is the Primary Predictor for Translocation Breakpoints

Above, we found that translocation breakpoints preferentially occur in chromosomal regions that are longer and harbor more open chromatin, more CTCF/cohesin binding sites and more genes. Importantly, these factors are often associated with each other [32]. Multiple (linear) regression analysis is often applied to test the individual contributions of multiple, potentially dependent, factors [37]. Thus, we performed multiple regression analysis to investigate which parameters are most significantly associated with translocation breakpoints. In human blood tumors, the length of cytogenetic bands, chromatin density and CTCF/cohesin binding site density showed significant contribution to the multiple regression model (all *p* < 0.01; Table 1). However, gene density did not. We also performed the multiple regression test on data excluding highly recurrent outlier translocations. Interestingly, CTCF/cohesin binding site density also no longer contributed significantly. This suggests that the likelihood for translocation breakpoints increases more readily by cytogenetic band length and chromatin density than by CTCF/cohesin binding site or gene density (Table 1).

We next analyzed the parameters in blood cancers using a multiple regression model with length-adjusted translocation frequencies. Chromatin density and CTCF/cohesin binding site density were shown to significantly contribute to the model (*p* = 8.5 × 10^−8^ and *p* = 0.032, respectively). However, CTCF/cohesin binding site density no longer did after we excluded outliers (Table 1).

For solid cancers, we performed multiple regression analyses in the same way. This yielded similar results. In non-length-adjusted analyses, length and chromatin density strongly contributed to the model irrespective of whether outliers were removed (all *p* < 0.0001; Table 1). However, after length-adjustment, only chromatin density significantly contributed to the model (*p* = 1 × 10^−9^; Table 1).

Taken together, we identify chromatin density as the primary predictor for translocation breakpoints in both blood and solid tumors. Our data indicate that translocation breakpoints preferentially occur in loosely packed chromatin.

### 2.6. Translocation Breakpoints Preferentially Occur in Acrocentric Chromosome Arms

**Figure 5:**
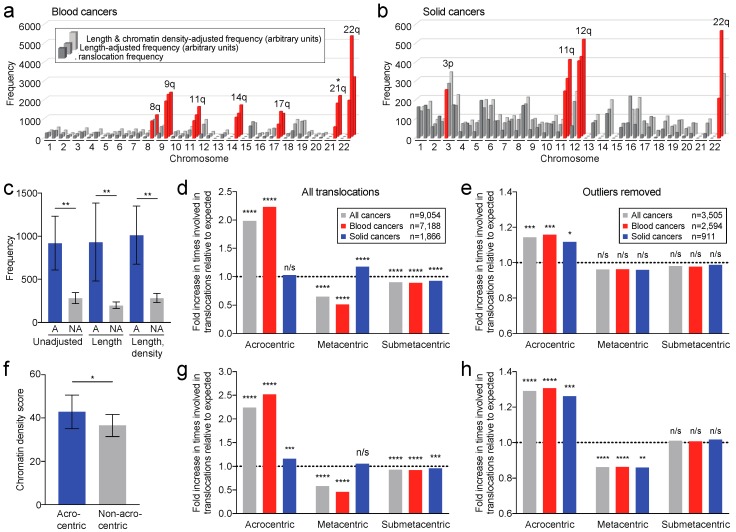
Translocation breakpoints preferentially occur in acrocentric chromosomes. (**a**) Bar graph showing the frequencies of translocation breakpoints per chromosome arm in blood cancers. From the front to the back, respectively, bars show translocation frequencies, chromosome arm length-adjusted frequencies (arbitrary units) and frequencies adjusted for both chromosome arm length and chromatin density (arbitrary units). Bars in red highlight statistically significantly increased frequencies compared to the frequencies of all chromosome arms. After removal of outlier translocations, only the frequency of chromosome arm 21q remains statistically significantly increased (*). (**b**) Bar graph as in (**a**) for solid cancers. No chromosome arm remains statistically significantly more frequently involved in translocations after removal of outlier translocations. (**c**) Bar graph showing that acrocentric chromosome arms (A) are significantly more often involved in translocations than non-acrocentric chromosome arms (NA), even after accounting for their physical length or physical length and chromatin density combined. *p*-values: Mann-Whitney *U* test. (**d**) Bar graph showing the fold increase in the observed involvement of chromosomes in translocations compared to the expected frequencies, which takes into account the length of the chromosomes. *p*-values: binomial test. (**e**) Bar graph as in (**d**) but with statistically identified outlier translocations removed. (**f**) Bar graph comparing chromatin densities of acrocentric and non-acrocentric chromosomes. *p*-value: *t*-test. (**g**) Bar graph as in (**d**) but with expected frequencies adjusted for different chromatin densities of the chromosomes. (**h**) Bar graph as in (**g**) but with statistically identified outlier translocations removed. N/s, not significant; *, *p* < 0.05; **, *p* < 0.01; ***, *p* < 0.001; ****, *p* < 0.0001.

We next determined the translocation frequencies for each chromosome arm—irrespective of their translocation partner—to identify specific arms that are recurrently involved in translocations. In blood cancers, seven chromosome arms are involved in translocations at significantly increased frequencies compared to the frequencies of all other arms (ROUT test at FDR *q* = 0.01) (Figure 5a). Above, we found that translocations preferentially occur in longer cytogenetic bands, or longer chromosome arms, and open chromatin (Table 1). Following adjustment for chromosome arm length or arm length and chromatin density, this finding remained largely unchanged (Figure 5a). However, removal of outlier translocations had a considerable impact, leaving only translocations in chromosome arm 21q as significantly recurrent (ROUT test at *q* = 0.01) (Figure 5a). Similar analyses for solid cancers identified translocations in two to four arms as significantly recurrent, yet none of these remained significant following removal of highly frequent outlier translocations (ROUT test at *q* = 0.01) (Figure 5b).

Strikingly, the data in Figure 5a suggested that acrocentric chromosome arms are preferentially involved in translocations, even though they are typically shorter. Indeed, the average translocation frequency in acrocentric chromosome arms was significantly higher than that average for non-acrocentric chromosome arms (*p* = 0.0027; Mann-Whitney *U* test) (Figure 5c) and this difference remained highly significant after adjusting for arm length or arm length and chromatin density (*p* = 0.0081, *p* = 0.0061, respectively) (Figure 5c). Thus, we conclude that translocations preferentially occur in acrocentric chromosome arms.

### 2.7. Translocation Breakpoints Preferentially Occur in Acrocentric Chromosomes

We wondered whether chromosome translocations preferentially occur in metacentric, submetacentric and/or acrocentric whole-chromosomes. To assess this, we calculated the expected percentages at which each of these types of chromosomes would be involved in translocations, taking into account the fraction of the cumulative length of each chromosome type within the whole genome (see Methods). Next, we compared these expected frequencies to our observed rates. We found that acrocentric chromosomes are involved in translocations nearly twice as often as expected (*p* < 0.0001; binomial test), while metacentric and submetacentric chromosomes are significantly less often involved in translocations (*p* < 0.0001; Figure 5d). Blood cancers predominantly contribute to this phenomenon, as they show a 2.2-fold higher than expected involvement of acrocentric chromosomes (*p* < 0.0001; Figure 5d). In solid cancers, acrocentric chromosomes are also more often involved in translocations than expected, but this increase is not statistically significant (*p* = 0.2414; Figure 5d).

It is possible that these observations are skewed due to the contribution of one or several translocations that occur at very high frequencies and hence represent outliers. However, after removal of outliers (using the ROUT test at FDR *q* = 0.01), acrocentric chromosomes still showed a significantly higher than expected involvement in translocations (*p* < 0.001) (Figure 5e). In fact, in solid cancers acrocentric chromosomes were now also significantly more often than expected involved in translocations (*p* = 0.0394), indicating that outlier translocations, in particular involving metacentric chromosomes, introduced a bias that masked preferential involvement of acrocentric chromosomes in these cancers (Figure 5d,e). Thus, we conclude that translocation breakpoints preferentially occur in acrocentric chromosomes in both hematological and solid cancers.

We compared this observation to translocations included in the “*Atlas of Genetics and Cytogenetics in Oncology and Haematology*” [38] (Table S1). However, this resource only lists unique translocations that have been reported in the literature. It notably does not include translocation frequencies. Hence, this precluded direct comparison of our observations to those in this Atlas (Table S1).

Above, we found that translocations preferentially occur in open chromatin and our multiple regression analysis indicated that chromatin density is the primary predictor for the occurrence of translocation breakpoints. Thus, if acrocentric chromosomes have more open chromatin—or a lower chromatin density score—then that could explain why they are more often involved in translocations. To test this, we compared the chromatin density scores of acrocentric chromosomes to those of non-acrocentric chromosomes. This indicated that acrocentric chromosomes are in fact significantly more chromatin-dense than non-acrocentric chromosomes (*p* = 0.0418; *t*-test) (Figure 5f). Even if this figure would not have shown statistical significance, chromatin density could skew analyses on a per-chromosome basis. However, the current observation means that our data in Figure 5d,e are an underestimation and that acrocentric chromosomes are preferentially involved in translocations despite the fact that they are more chromatin-dense.

To account for the more heterochromatic state of acrocentric chromosomes, we adjusted the expected rates at which the chromosome types are involved in translocations to their respective chromatin densities. This showed that in both blood and solid cancers, acrocentric chromosomes are preferentially involved in translocations (all *p* values < 0.001) (Figure 5g), irrespective of whether outliers are removed (all *p* values < 0.001) (Figure 5h). Our data also strongly suggest that this occurs mostly at the expense of translocations involving metacentric chromosomes (Figure 5d,e,g,h).

### 2.8. Identification of Significantly Recurrent and Underrepresented Translocations

A considerable number of highly recurrent translocations—including the respective fusion genes—have previously been identified [15,38]. In addition to these, we here aim to identify less common translocations, as well as significantly underrepresented translocations, as the latter could reveal strong negative selection. To do so, we used a previously described probabilistic model developed to identify statistically significant pair-wise patterns of species co-occurrence [39]. Each translocation can be considered a co-occurring pair of chromosome arms or chromosome bands. Accordingly, we performed a co-occurrence analysis for translocations in blood and solid tumors.

**Figure 6:**
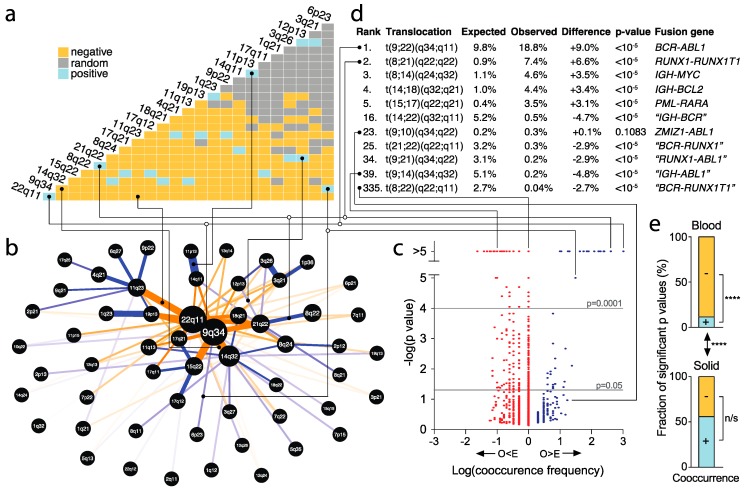
Identification of significantly recurrent and underrepresented translocations. (**a**) Matrix of co-occurring pairs of breakpoints in the indicated cytogenetic chromosome bands, which constitute translocations. For each pair/translocation, *p*-values were calculated using Veech’s probabilistic model [39]. Statistically significantly (*p* < 0.05) more observed than expected (“positive”) translocations—taking into account the frequencies at which each individual cytogenetic chromosome band is involved in translocations—are shown in blue. Translocations occurring at significantly lower than expected frequencies (“negative”) are shown in orange. Non-statistically significant pairs (“random”) are shown in grey. Only part of the matrix is shown. The full matrix is shown in Supplementary Figure S1a. (**b**) Network of translocations in blood cancers per cytogenetic chromosome band (see also Supplementary Figure S1a). Each node represents a cytogenetic band. Node size is proportional to the frequency at which translocation breakpoints occur in the band. Edges represent the specific translocations/co-occurring pairs. Thickness of the edges is inversely proportional to the *p*-value according to Veech’s probabilistic model. Blue edges indicate statistically significantly more observed than expected translocations. Orange edges indicate significantly lower than expected frequencies. Only the top 100 significant pairs are shown. (**c**) Volcano plot for translocations/co-occurrences. For each translocation, the –log(*p*-value), according to Veech’s probabilistic model, is plotted against its frequency. Frequencies are shown as negative, if the observed frequency (O) is smaller than the expected frequency (E) and as positive if O > E. See also Figure S2. (**d**) Table of the top five most common and selected other translocations in blood cancers. The full table is shown in Supplementary Table S2. Lines link selected translocations in (**a**–**d**). (**e**) Distribution of the numbers of statistically significant positive and negative translocations in blood and solid cancers. *p*-values: Fisher’s exact tests. N/s, not significant; ****, *p* < 0.0001.

We built matrices for the co-occurrences/translocations. Statistically significant pairs were identified based on *p*-values smaller than 0.05 (Veech’s probabilistic model [39]). At the chromosome arm level, there were 299 significant pairs in blood tumors, compared to 133 pairs in solid tumors, whereas at the cytogenetic chromosome band level, we identified 298 significant pairs for blood tumors and 25 significant over- or underrepresented pairs for solid tumors (Figure 6a, Supplementary Figure S1, Tables S2–S5). Next, to better visualize the co-occurrences, we generated networks of the most frequent co-occurrences (Figure 6b, Supplementary Figure S1), as well as volcano plots, which included all translocations (Figure 6c, Supplementary Figure S2). This led to a number of observations.

First, consistent with previous findings, these analyses indicate that chromosomal translocations are much more prevalent in blood tumors than in solid cancers (Figure 6, Supplementary Figure S1).

Second, not surprisingly, with 18.8% and 7.4% of all translocations, t(9;22)(q34;q11) and t(8;21)(q22;q22)—corresponding to the Philadelphia chromosome/*BCR*-*ABL1* and *RUNX1*-*RUNX1T1*/*AML1-ETO* fusion genes [12,15]—were the most frequent translocations in blood cancers (Figure 6d, Supplementary Figure S1, Table S2). Strikingly, however, the highly prevalent occurrence of translocation breakpoints at these and several other locations predicted fusion genes such as “*IGH*-*BCR*”, “*BCR*-*RUNX1*”, “*RUNX1*-*ABL1*”, “*IGH*-*ABL1*” and “*BCR*-*RUNX1T1*” at frequencies up to 5.2%, which would have represented the third most common translocation in blood cancers (Figure 6d, Supplementary Table S2). However, these were identified at significantly lower than expected frequencies, ranging from only 0.04% to 0.5% (all *p* < 0.00001) (Figure 6d, Table S2). This strongly suggests that such translocations do not provide a survival advantage for hematological cancer cells or that there is strong negative selection against them.

Third, similarly, in solid cancers, the most frequent translocations were t(11;22)(q24;q12) and t(12;16)(q13;p11), which correspond to *EWSR1*-*FLI1* and *FUS*-*ATF1* fusion genes, frequently found in Ewing’s sarcoma and myxoid or round cell liposarcoma, respectively (Table S3) [15]. Translocation t(12;22)(q13;q12), with “*ATF1*-*EWSR1*” as a predicted fusion gene, was expected at nearly 1% of translocations—which would have ranked sixth most common in solid cancers. However, this translocation occurred in only one patient (0.07%, ranking 76th), significantly lower than expected (*p* = 0.00001), thus also suggesting a lack of survival advantage or strong negative selection against it during tumorigenesis.

Finally, for solid cancers, the numbers of significant positive and negative correlation pairs were about equally distributed. However, we observed a considerably lower number of significant positive than significant negative correlation pairs in blood cancers (*p* < 0.0001, Fisher’s exact test; Figure 6a,c,e, Supplementary Figure S1, Tables S2 and S3). Yet, a small number of the most frequent translocations in blood cancers showed the strongest significance. Notably, the top four most frequent translocations represented more than a third of all translocations and the top ten constituted half of all translocations (Supplementary Table S2). This strongly suggests that blood cancer cells with few specific translocations harbor considerable malignant advantages that provide benefits for tumorigenesis.

## 3. Discussion

Chromosomal translocations have been shown to promote tumorigenesis in many types of cancer, including leukemia [12], lymphoma [13], sarcoma [14], breast carcinoma [40] and lung carcinoma [15,41]. A variety of mechanisms have been proposed to underlie chromosomal translocations, involving both the generation of DNA DSBs and the fusion of breakpoint sites on heterologous chromosomes [42,43]. These relate to V(D)J recombination, gene expression and chromatin density. However, where in the genome the translocation breakpoints are most likely to occur remains incompletely understood. This may in part be due to interdependencies of proposed factors (see also below). Here, using 1563 karyotypes of solid tumors and 13,844 karyotypes of blood tumors, we assessed the associations of several parameters with the frequency of translocation breakpoints in blood and solid tumors..

We find that translocations more often occur in longer cytogenetic chromosome bands. While this might have a biological cause, we believe that this simply reflects an increased mathematical probability. Hence, we also performed our analyses on chromosome band length-adjusted translocation frequencies.

Transcription has been linked to genome instability. It may alter the DNA sequence or promote chromosomal rearrangement [44]. Using genome-wide translocation sequencing to analyze DSBs as translocation hotspots, two comprehensive studies found that translocations are strongly associated with transcription start sites in the genome [45,46]. These observations are consistent with our finding that translocation breakpoints occur more frequently in regions enriched in genes and CTCF/cohesin binding sites, the latter of which are important for both transcription and enhancer-promoter interactions [28,32].

Chromatin density has also previously been linked to influence translocation frequency [42,47]. Open chromatin is thought to be more susceptible to DNA DSBs than heterochromatin, as the latter is protected by proteins that mediate higher order chromatin condensation and DSBs are the first requirement for the generation of translocations. Consistent with this thesis, we also find that translocation breakpoints preferentially occur in open chromatin.

Adding to the complexity of identifying which factors promote translocations, a number of parameters are often associated with each other. For example, chromatin density, gene density and CTCF/cohesin binding site density are linked. After all, CTCF/cohesin affects transcriptional activity and chromatin density [32], open chromatin is required for transcription and transcription occurs where genes are located. Importantly, however, multiple regression analysis enabled us take such interdependencies into account. This indicated that chromatin density is a more significant predictor for translocation breakpoints than CTCF/cohesin binding site density or gene density.

We observed vastly distinct chromosomal translocation signatures in blood and solid tumors. This may be attributed to the profound differences between hematological cell types and those of mesenchymal or epithelial origin, for example in chromosomal organization or dynamics [48]. Some studies showed that the spatial proximity of heterologous loci undergoing DSBs promotes ligation—and hence the translocation—between them [48,49,50]. This phenomenon may partly explain why some translocations occur at extremely high frequencies.

More broadly, the forms of genomic instability that drive blood and solid tumorigenesis are also markedly different. Hematological cancer development is typically facilitated by the expression of fusion genes as a result of translocations [11,15]. In contrast, solid tumorigenesis is more often promoted by common aberrations in tumor suppressor pathways, which in turn lead to whole-chromosome instability or forms of structural chromosome instability that may or may not include translocations [1,5,6,51].

We identified acrocentrism as a novel chromosomal attribute that predisposes to translocations. In blood tumors, nearly a third of all translocations involve acrocentric chromosomes. After removal of highly prevalent translocations, this observation remained highly statistically significant. In contrast, acrocentric chromosomes were also more frequently than expected involved in solid cancer translocations. However, this increase was statistically significant only in multivariate analyses or after removal of outliers.

Our observations provide insights into where translocations may be generated subcellularly. Within the nucleus, chromosomes are organized in territories [52,53]. Also, the short arms of acrocentric chromosomes harbor ribosomal DNA, which is organized in nucleolar organiser regions (NORs) [54]. These NORs—and hence the short arms of acrocentric chromosomes—consistently localize to nucleoli, which are located in the inner nuclear space, rather than at the nuclear periphery. Consistently, acrocentric chromosomes localize to the core of the nucleoplasm, away from the nuclear lamina, where larger chromosomes in particular are located [53]. Thus, our finding that translocations preferentially occur in acrocentric chromosomes suggests that cancerous chromosomal translocations are often generated perinucleolarly, in the inner nuclear space, away from the nuclear lamina.

Interestingly, translocation breakpoints in the germline often overlap with those in tumors [55]. Consistent with this, germline translocations also frequently involve acrocentric chromosomes. In fact, almost all translocations in the germline are Robertsonian translocations, involving two acrocentric chromosomes. For example, the Robertsonian translocation between chromosomes 14 and 21 is sometimes detected in the germline [56]. Offspring of such translocation carriers may be trisomic for chromosome 21 and affected by Down syndrome.

Our pairwise probabilistic co-occurrence modeling identified highly significant translocations involving chromosome 9q34 and 22q11, chromosome 8q24 and 14q32, as well as chromosome 15q22 and 17q21. It is well documented that the formation of fusion oncogenes from those translocations, including *ABL1* and *BCR* [57], *MYC* [58], *PML* and *RARA* [59], cause blood tumorigenesis [11,15]. Yet, our identification of significantly underrepresented translocations and putative fusion genes is novel and suggests that these are probably strongly selected against during tumor evolution.

Lastly, the generation of “fusion mRNAs” has also been proposed as a potential tumorigenic mechanism [60]. Such fusion mRNAs are generated from early-terminated transcripts, rather than from rearranged genomic loci. This suggests that malignant fusion transcripts may be more common in cancer cells than expected based on translocation frequencies alone.

Taken together, we find that cancerous translocations preferentially occur in euchromatin and acrocentric chromosomes. Probabilistic co-occurrence modeling identified well-known recurrent translocations, as well as markedly underrepresented translocations, which either do not provide proliferative advantages, or against which strong negative selection occurs during tumor progression. Thus, our findings generated novel insights into the mechanisms and selection of translocations during tumorigenesis.

## 4. Materials and Methods

### 4.1. Karyotype Selection and Translocation Frequencies

Karyotypes from human tumors were collected from the Mitelman Database of Chromosome Aberrations in Cancer [61]. Biases in the Mitelman database karyotypes were previously reported [35]. Hence, our analyses only included quality-checked karyotypes, as described by Ozery-Flato and colleagues [35]. A total of 1563 karyotypes from solid tumors and 13,844 karyotypes from blood cancers were analyzed (also available from the “STACK” database at http://acgt.cs.tau.ac.il/stack [35]). Translocation frequencies within each cytogenetic chromosome band, chromosome arm or whole chromosome were determined using these data.

### 4.2. Definitions of Parameters

Physical length of cytogenetic chromosome bands, chromosome arms or whole chromosomes, as well as the numbers of genes and CTCF/cohesin DNA binding sites in each of these were obtained from the Human Genome Browser (https://genome.ucsc.edu), University of California Santa Cruz (Santa Cruz, CA, USA). Gene density and CTCF/cohesin binding site density were calculated by dividing the number of genes or number of CTCF/cohesin binding sites in each cytogenetic band by the physical length of the cytogenetic band. The average chromatin density (ACD) score was calculated using the intensities of Giemsa staining of each chromosome band, as depicted in shades of grey in the conventional ideogram, ranging from white (euchromatin, score 0) to black (heterochromatin, score 100). Each chromosome sub-band received a discrete score of 0, 25, 50, 75, or 100. The ACD score of each chromosome band was calculated as the weighted average of the discrete scores of each sub-band (taking into account the length/proportion of each chromosome sub-band within the cytogenetic band). To which cytogenetic type (i.e., acrocentric, sub-metacentric or metacentric) each chromosome belonged was determined by the position of the centromere within the chromosome.

### 4.3. Statistical Analyses

#### 4.3.1. Data Distributions, Outliers and Linear Regression

Statistical assessment of translocation frequencies, cytogenetic band lengths, ACD scores, CTCF/cohesin binding site densities and gene densities was performed as described [62,63]. D'Agostino-Pearson normality tests showed that none of these parameters were normally distributed (all *p* < 0.0001). The non-Gaussian distribution of the translocation frequencies, as well as the observation of several extremely high translocation frequencies within the dataset, prompted us to identify outliers. Where indicated, data were re-analyzed after outlier removal to ensure that conclusions were not reached mostly or exclusively due to the strong contributions of outliers. In all analyses, outliers were identified using the ROUT method [36] with a false discovery rate of *q* = 0.01 using GraphPad Prism software (GraphPad Software, Inc., La Jolla, CA, USA). In order to make highly skewed distributions less skewed, translocation frequency data were log_10_-transformed. Spearman’s rank-order correlations were used to assess the extent to which parameters associated with chromosome translocation frequency. Spearman regression coefficients (*r*) were presented to assess the relationship between translocation frequencies and factors of interest. The *p* values express the probability that the observed value was not due to chance.

#### 4.3.2. Multiple Regression Analysis

Multiple regression analysis was used to determine the contribution of each variable to the chromosomal translocations. Regression coefficients of each predictor indicated the mean change in the translocation for one unit of change in the predicted factor while holding other factors of interest in the model constant. The goodness-of-fit of the multiple linear regression for the model was expressed by *R*^2^ and adjusted *R*^2^ values. The *p* values express the probability that the slope of the multiple linear regression line is zero.

#### 4.3.3. Chromosome Type Analyses

For chromosome arms, absolute translocation frequencies were presented. Adjustment to compensate for arm length occurred by dividing the absolute translocation frequencies by the respective physical chromosome arm lengths. Additional adjustment to account for chromatin density occurred by multiplying the latter by the respective ACD scores. For whole chromosomes, absolute and adjusted translocation frequencies were calculated similarly.

The expected rates at which translocations occur in each of the chromosome types, i.e., $E_{type}$, referring to $E_{acrocentric}$, $E_{metacentric}$ and $E_{submetacentric}$, were calculated using Equation (1).

(1) $$E_{type}=O_{total}\times \frac{n_{type}}{n_{chromosome}}$$

Herein, $O_{total}$ is the observed total translocation frequency and n the number of chromosomes in the group. Subscript “*chromosome*” refers to any chromosome. The fact that the genome is diploid is accounted for in Equation (1). Expected frequencies adjusted to physical chromosome length (l) were computed according to Equation (2).

(2) $$E_{type}=O_{total}\times \frac{2\times \sum l_{type}}{2\times \sum l_{chromosome}}$$

Additional adjustment to chromatin density occurred according to Equation (3).

(3) $$E_{type}=O_{total}\times \frac{2\times \sum (l_{type}\times d_{type})}{2\times \sum (l_{chromosome}\times d_{chromosome})}$$

Herein, d refers to the ACD score. To determine whether observed frequencies were statistically significantly different from expected frequencies, binomial tests were applied. GraphPad Prism software was used to determine the *p* values. Abbreviations used were: n/s, not significant; *, *p* < 0.05; **, *p* < 0.01; ***, *p* < 0.001; ****, *p* < 0.0001.

#### 4.3.4. Probabilistic Co-Occurrence Modeling

Pairwise probabilistic co-occurrence modeling, or Veech’s probabilistic modeling, was performed as previously described [39]. Briefly, each translocation was considered the co-occurrence of two chromosomal breakpoints. Observed co-occurrence frequencies $O_{cooccur}$, calculation of expected co-occurrence frequencies $E_{cooccur}$ (based on the frequencies of the individual breakpoints) computation of the *p* values, reflecting the probability that $O_{cooccur}$ > $E_{cooccur}$ (“positive” co-occurrence) or $O_{cooccur}$ < $E_{cooccur}$ (“negative” co-occurrence) occurred by chance, and generation of matrices, networks and volcano plots were performed in the *R* programming environment.

## 5. Conclusions

The existence of chromosomal translocations in human tumors has been known for many decades [15]. More recently, a number of factors have been identified that influence where in the genome translocation breakpoints occur. These include chromatin density, gene density and CTCF)/cohesin binding site density. However, interdependence of these factors has considerably complicated deciphering the precise contribution of each of these. Our multiple linear regression analyses on thousands of blood and solid cancers identified chromatin density as the primary contributor with breakpoints preferentially occurring in open chromatin. We also identified acrocentrism as a novel predisposing factor. As the short arms of acrocentric chromosomes localize to the nucleoli, this suggests that translocations are often generated around nucleoli and hence in the inner nucleoplasm, rather than close to the nuclear envelope. Using pairwise probabilistic co-occurrence modeling, we identified both highly prevalent and significantly underrepresented translocations with putative fusion genes. The latter are probably strongly selected against during tumor development. Thus, our discoveries have shed new light on both the generation and selection of translocations during tumorigenesis.

## Figures and Tables

**Table 1:** Multiple regression analysis for translocation breakpoints in human cancers.

Model	Blood Cancers				Solid Cancers			
	Non-Adjusted							
**Variable**	***B***	**Standard Error**	***t* Value**	***p* Value**	***B***	**Standard Error**	***t* Value**	***p* Value**
Length	0.0280 (0.0168)	0.0046 (0.0038)	5.97 (4.39)	6.6 × 10^−9^ (1.6 × 10^−5^)	0.0171 (0.0164)	0.0043 (0.0034)	3.96 (4.80)	9.4 × 10^−5^ (2.74 × 10^−6^)
Chromatin Density Score	−0.0041 (−0.0034)	0.0008 (0.0006)	−5.20 (−5.74)	3.7 × 10^−7^ (2.57 × 10^−8^)	−0.0047 (−0.0044)	0.0007 (0.0006)	−6.34 (−7.56)	8.9 × 10^−10^ (6.91 × 10^−13^)
CTCF/Cohesin Density	0.0116 (0.0060)	0.0039 (0.0031)	2.93 (1.97)	0.003 (0.0504)	1.77 × 10^−5^ (−0.0005)	0.0036 (0.0029)	0.005 (−0.19)	0.996 (0.849)
Gene Density	0.0146 (0.0108)	0.0076 (0.0066)	1.91 (1.62)	0.056 (0.107)	0.0114 (0.0066)	0.0070 (0.0058)	1.63 (1.14)	0.105 (0.255)
**Model Summary**	*N* = 292, *R*^2^ = 0.40, adjusted *R*^2^ = 0.39, *p* < 0.001 (*N* = 259, *R*^2^ = 0.32, adjusted *R*^2^ = 0.31, *p* < 0.001)				*N* = 292, *R*^2^ = 0.24, adjusted *R*^2^ = 0.23, *p* < 0.001 (*N* = 265, *R*^2^ = 0.29, adjusted *R*^2^ = 0.28, *p* < 0.001)			
	**Length-Adjusted**							
**Variable**	***B***	**Standard Error**	***t* Value**	***p* Value**	***B***	**Standard Error**	***t* Value**	***p* Value**
Chromatin Density Score	−0.0033 (−0.0021)	0.0006 (0.0003)	−5.50 (−5.79)	8.5 × 10^−8^ (2.08 × 10^−8^)	−0.0031 (−0.0018)	0.0004 (0.0003)	−7.11 (−6.34)	9.4 × 10^−12^ (1 × 10^−9^)
CTCF/Cohesin Density	0.0065 (0.0027)	0.0030 (0.0018)	2.15 (1.46)	0.032 (0.147)	0.0029 (0.0004)	0.0022 (0.0014)	−1.31 (0.28)	0.191 (0.779)
Gene Density	0.0102 (0.0073)	0.0059 (0.0039)	1.74 (1.90)	0.083 (0.058)	0.0062 (0.0025)	0.0043 (0.0027)	1.45 (0.91)	0.149 (0.364)
**Model Summary**	*N* = 292, *R*^2^ = 0.293, adjusted *R*^2^ = 0.286, *p* < 0.001 (*N* = 257, *R*^2^ = 0.302, adjusted *R*^2^ = 0.293, *p* < 0.001)				*N* = 292, *R*^2^ = 0.187, adjusted *R*^2^ = 0.179, *p* < 0.001 (*N* = 266, *R*^2^ = 0.223, adjusted *R*^2^ = 0.215, *p* < 0.001)			

CTCF, CCCTC-binding factor; Data shown in parentheses represent values after removal of outliers (see main text and Methods for details).

## Supplementary Materials

The following are available online at http://www.mdpi.com/2072-6694/10/1/13/s1. Figure S1: Matrices and networks of translocation/co-occurrence analyses, Figure S2: Volcano plots for translocation/co-occurrence analyses, Table S1: Numbers of unique translocations listed in the Atlas of Genetics and Cytogenetics in Oncology and Haematology, Table S2: Veech’s probabilistic model of translocations in blood cancers at the cytogenetic chromosome band level, Table S3: Veech’s probabilistic model of translocations in solid cancers at the cytogenetic chromosome band level, Table S4: Veech’s probabilistic model of translocations in blood cancers at the chromosome arm level, Table S5: Veech’s probabilistic model of translocations in solid cancers at the chromosome arm level.

## References

[1] Negrini S., Gorgoulis V.G., Halazonetis T.D. (2010). Genomic instability—An evolving hallmark of cancer. Nat. Rev. Mol. Cell Biol..

[2] Tanaka K., Hirota T. (2016). Chromosomal instability: A common feature and a therapeutic target of cancer. Biochim. Biophys. Acta.

[3] Habermann J.K., Doering J., Hautaniemi S., Roblick U.J., Bundgen N.K., Nicorici D., Kronenwett U., Rathnagiriswaran S., Mettu R.K., Ma Y. (2009). The gene expression signature of genomic instability in breast cancer is an independent predictor of clinical outcome. Int. J. Cancer.

[4] Thompson L.L., Jeusset L.M., Lepage C.C., McManus K.J. (2017). Evolving therapeutic strategies to exploit chromosome instability in cancer. Cancers.

[5] Duijf P.H., Schultz N., Benezra R. (2013). Cancer cells preferentially lose small chromosomes. Int. J. Cancer.

[6] Duijf P.H., Benezra R. (2013). The cancer biology of whole-chromosome instability. Oncogene.

[7] Van Jaarsveld R.H., Kops G.J. (2016). Difference makers: Chromosomal instability versus aneuploidy in cancer. Trends Cancer.

[8] Ghadimi B.M., Sackett D.L., Difilippantonio M.J., Schrock E., Neumann T., Jauho A., Auer G., Ried T. (2000). Centrosome amplification and instability occurs exclusively in aneuploid, but not in diploid colorectal cancer cell lines, and correlates with numerical chromosomal aberrations. Genes Chromosomes Cancer.

[9] Vaidyanathan S., Cato K., Tang L., Pavey S., Haass N.K., Gabrielli B.G., Duijf P.H. (2016). In vivo overexpression of emi1 promotes chromosome instability and tumorigenesis. Oncogene.

[10] Thangavelu P.U., Lin C.Y., Vaidyanathan S., Nguyen T.H.M., Dray E., Duijf P.H.G. (2017). Overexpression of the E2F target gene CENPI promotes chromosome instability and predicts poor prognosis in estrogen receptor-positive breast cancer. Oncotarget.

[11] Zheng J. (2013). Oncogenic chromosomal translocations and human cancer (review). Oncol. Rep..

[12] Nowell P.C. (2007). Discovery of the Philadelphia chromosome: A personal perspective. J. Clin. Invest..

[13] Chaganti S.R., Chen W., Parsa N., Offit K., Louie D.C., Dalla-Favera R., Chaganti R.S. (1998). Involvement of BCL6 in chromosomal aberrations affecting band 3q27 in B-cell non-Hodgkin lymphoma. Genes Chromosomes Cancer.

[14] Kim S.K., Park Y.K. (2016). Ewing sarcoma: A chronicle of molecular pathogenesis. Hum. Pathol..

[15] Nambiar M., Kari V., Raghavan S.C. (2008). Chromosomal translocations in cancer. Biochim. Biophys. Acta.

[16] Salmon J.M., Bots M., Vidacs E., Stanley K.L., Atadja P., Zuber J., Johnstone R.W. (2015). Combining the differentiating effect of panobinostat with the apoptotic effect of arsenic trioxide leads to significant survival benefit in a model of t(8;21) acute myeloid leukemia. Clin. Epigenetics.

[17] Guo G., Kang Q., Zhu X., Chen Q., Wang X., Chen Y., Ouyang J., Zhang L., Tan H., Chen R. (2015). A long noncoding RNA critically regulates Bcr-Abl-mediated cellular transformation by acting as a competitive endogenous RNA. Oncogene.

[18] Chapman J.R., Taylor M.R., Boulton S.J. (2012). Playing the end game: DNA double-strand break repair pathway choice. Mol. Cell.

[19] Janssen A., van der Burg M., Szuhai K., Kops G.J., Medema R.H. (2011). Chromosome segregation errors as a cause of DNA damage and structural chromosome aberrations. Science.

[20] Weiler K.S., Wakimoto B.T. (1995). Heterochromatin and gene expression in drosophila. Annu. Rev. Genet..

[21] Schatz D.G., Baltimore D. (2004). Uncovering the V(D)J recombinase. Cell.

[22] Fugmann S.D., Lee A.I., Shockett P.E., Villey I.J., Schatz D.G. (2000). The RAG proteins and V(D)J recombination: Complexes, ends, and transposition. Annu. Rev. Immunol..

[23] Lieber M.R. (2010). The mechanism of double-strand DNA break repair by the nonhomologous DNA end-joining pathway. Annu. Rev. Biochem..

[24] Raghavan S.C., Kirsch I.R., Lieber M.R. (2001). Analysis of the V(D)J recombination efficiency at lymphoid chromosomal translocation breakpoints. J. Biol. Chem..

[25] Marculescu R., Le T., Simon P., Jaeger U., Nadel B. (2002). V(D)J-mediated translocations in lymphoid neoplasms: A functional assessment of genomic instability by cryptic sites. J. Exp. Med..

[26] Rooney S., Alt F.W., Lombard D., Whitlow S., Eckersdorff M., Fleming J., Fugmann S., Ferguson D.O., Schatz D.G., Sekiguchi J. (2003). Defective DNA repair and increased genomic instability in artemis-deficient murine cells. J. Exp. Med..

[27] Guo C., Yoon H.S., Franklin A., Jain S., Ebert A., Cheng H.L., Hansen E., Despo O., Bossen C., Vettermann C. (2011). CTCF-binding elements mediate control of V(D)J recombination. Nature.

[28] Ong C.T., Corces V.G. (2014). CTCF: An architectural protein bridging genome topology and function. Nat. Rev. Genet..

[29] Parelho V., Hadjur S., Spivakov M., Leleu M., Sauer S., Gregson H.C., Jarmuz A., Canzonetta C., Webster Z., Nesterova T. (2008). Cohesins functionally associate with CTCF on mammalian chromosome arms. Cell.

[30] Rubio E.D., Reiss D.J., Welcsh P.L., Disteche C.M., Filippova G.N., Baliga N.S., Aebersold R., Ranish J.A., Krumm A. (2008). CTCF physically links cohesin to chromatin. Proc. Natl. Acad. Sci. USA.

[31] Wendt K.S., Yoshida K., Itoh T., Bando M., Koch B., Schirghuber E., Tsutsumi S., Nagae G., Ishihara K., Mishiro T. (2008). Cohesin mediates transcriptional insulation by CCCTC-binding factor. Nature.

[32] Herold M., Bartkuhn M., Renkawitz R. (2012). CTCF: Insights into insulator function during development. Development.

[33] Howarth K.D., Blood K.A., Ng B.L., Beavis J.C., Chua Y., Cooke S.L., Raby S., Ichimura K., Collins V.P., Carter N.P. (2008). Array painting reveals a high frequency of balanced translocations in breast cancer cell lines that break in cancer-relevant genes. Oncogene.

[34] Bates S.E. (2011). Classical cytogenetics: Karyotyping techniques. Methods Mol. Biol..

[35] Ozery-Flato M., Linhart C., Trakhtenbrot L., Izraeli S., Shamir R. (2011). Large-scale analysis of chromosomal aberrations in cancer karyotypes reveals two distinct paths to aneuploidy. Genome Biol..

[36] Motulsky H.J., Brown R.E. (2006). Detecting outliers when fitting data with nonlinear regression—a new method based on robust nonlinear regression and the false discovery rate. BMC Bioinformatics.

[37] Straughen J.K., Sipahi L., Uddin M., Misra D.P., Misra V.K. (2015). Racial differences in IGF1 methylation and birth weight. Clin. Epigenetics.

[38] Huret J.L., Ahmad M., Arsaban M., Bernheim A., Cigna J., Desangles F., Guignard J.C., Jacquemot-Perbal M.C., Labarussias M., Leberre V. (2013). Atlas of genetics and cytogenetics in oncology and haematology in 2013. Nucleic Acids Res..

[39] Veech J.A. (2013). A probabilistic model for analysing species co-occurrence. Global Ecol. Biogeogr..

[40] Tognon C., Knezevich S.R., Huntsman D., Roskelley C.D., Melnyk N., Mathers J.A., Becker L., Carneiro F., MacPherson N., Horsman D. (2002). Expression of the *ETV6-NTRK3* gene fusion as a primary event in human secretory breast carcinoma. Cancer Cell.

[41] Soda M., Choi Y.L., Enomoto M., Takada S., Yamashita Y., Ishikawa S., Fujiwara S., Watanabe H., Kurashina K., Hatanaka H. (2007). Identification of the transforming EML4-ALK fusion gene in non-small-cell lung cancer. Nature.

[42] Iarovaia O.V., Rubtsov M., Ioudinkova E., Tsfasman T., Razin S.V., Vassetzky Y.S. (2014). Dynamics of double strand breaks and chromosomal translocations. Mol. Cancer.

[43] Mills K.D., Ferguson D.O., Alt F.W. (2003). The role of DNA breaks in genomic instability and tumorigenesis. Immunol. Rev..

[44] Aguilera A. (2002). The connection between transcription and genomic instability. EMBO J..

[45] Klein I.A., Resch W., Jankovic M., Oliveira T., Yamane A., Nakahashi H., Di Virgilio M., Bothmer A., Nussenzweig A., Robbiani D.F. (2011). Translocation-capture sequencing reveals the extent and nature of chromosomal rearrangements in b lymphocytes. Cell.

[46] Chiarle R., Zhang Y., Frock R.L., Lewis S.M., Molinie B., Ho Y.J., Myers D.R., Choi V.W., Compagno M., Malkin D.J. (2011). Genome-wide translocation sequencing reveals mechanisms of chromosome breaks and rearrangements in B cells. Cell.

[47] Zhang Y., Gostissa M., Hildebrand D.G., Becker M.S., Boboila C., Chiarle R., Lewis S., Alt F.W. (2010). The role of mechanistic factors in promoting chromosomal translocations found in lymphoid and other cancers. Adv. Immunol..

[48] Roix J.J., McQueen P.G., Munson P.J., Parada L.A., Misteli T. (2003). Spatial proximity of translocation-prone gene loci in human lymphomas. Nat. Genet..

[49] Neves H., Ramos C., da Silva M.G., Parreira A., Parreira L. (1999). The nuclear topography of *ABL*, *BCR*, *PML*, and *RARα* genes: Evidence for gene proximity in specific phases of the cell cycle and stages of hematopoietic differentiation. Blood.

[50] Osborne C.S., Chakalova L., Mitchell J.A., Horton A., Wood A.L., Bolland D.J., Corcoran A.E., Fraser P. (2007). Myc dynamically and preferentially relocates to a transcription factory occupied by Igh. PLoS Biol..

[51] Schvartzman J.M., Duijf P.H., Sotillo R., Coker C., Benezra R. (2011). Mad2 is a critical mediator of the chromosome instability observed upon Rb and p53 pathway inhibition. Cancer Cell.

[52] Cremer T., Cremer M. (2010). Chromosome territories. Cold Spring Harb Perspect Biol..

[53] Bolzer A., Kreth G., Solovei I., Koehler D., Saracoglu K., Fauth C., Muller S., Eils R., Cremer C., Speicher M.R. (2005). Three-dimensional maps of all chromosomes in human male fibroblast nuclei and prometaphase rosettes. PLoS Biol..

[54] Henderson A.S., Warburton D., Atwood K.C. (1972). Location of ribosomal DNA in the human chromosome complement. Proc. Natl. Acad. Sci. USA.

[55] Van Heesch S., Simonis M., van Roosmalen M.J., Pillalamarri V., Brand H., Kuijk E.W., de Luca K.L., Lansu N., Braat A.K., Menelaou A. (2014). Genomic and functional overlap between somatic and germline chromosomal rearrangements. Cell Rep..

[56] Petersen M.B., Adelsberger P.A., Schinzel A.A., Binkert F., Hinkel G.K., Antonarakis S.E. (1991). Down syndrome due to de novo robertsonian translocation t(14q;21q): DNA polymorphism analysis suggests that the origin of the extra 21q is maternal. Am. J. Hum. Genet..

[57] De Klein A., van Kessel A.G., Grosveld G., Bartram C.R., Hagemeijer A., Bootsma D., Spurr N.K., Heisterkamp N., Groffen J., Stephenson J.R. (1982). A cellular oncogene is translocated to the philadelphia chromosome in chronic myelocytic leukaemia. Nature.

[58] Rabbitts T.H., Boehm T. (1991). Structural and functional chimerism results from chromosomal translocation in lymphoid tumors. Adv. Immunol..

[59] Borrow J., Goddard A.D., Sheer D., Solomon E. (1990). Molecular analysis of acute promyelocytic leukemia breakpoint cluster region on chromosome 17. Science.

[60] Kowarz E., Dingermann T., Marschalek R. (2012). Do non-genomically encoded fusion transcripts cause recurrent chromosomal translocations?. Cancers.

[61] Mitelman F., Johansson B., Mertens F.E. Mitelman Database of Chromosome Aberrations and Gene Fusions in Cancer. http://cgap.nci.nih.gov/Chromosomes/Mitelman.

[62] Thangavelu P.U., Krenacs T., Dray E., Duijf P.H. (2016). In epithelial cancers, aberrant COL17A1 promoter methylation predicts its misexpression and increased invasion. Clin. Epigenetics.

[63] Vaidyanathan S., Thangavelu P.U., Duijf P.H. (2016). Overexpression of ran gtpase components regulating nuclear export, but not mitotic spindle assembly, marks chromosome instability and poor prognosis in breast cancer. Target Oncol..

